# Multidisciplinary Surgical Management of Renal Cell Carcinoma With Inferior Vena Cava Tumor Thrombus: Perioperative and Oncological Outcomes

**DOI:** 10.7759/cureus.85527

**Published:** 2025-06-07

**Authors:** Ibrahim A Khalil, Majd Alkabbani, Mohamed Abdel-Latif, Nagy Younes, Alaeddin Badawi, Hassan Al-Thani, Khalid Al Rumaihi

**Affiliations:** 1 Department of Urology, Hamad Medical Corporation, Doha, QAT; 2 Department of Vascular and Trauma Surgery, Hamad General Hospital, Doha, QAT; 3 Department of Urology, College of Medicine, Qatar University, Doha, QAT

**Keywords:** inferior vena cava tumor thrombus, multidisciplinary team, radical nephrectomy, renal cell carcinoma, surgical oncology

## Abstract

Introduction

Renal cell carcinoma (RCC) with inferior vena cava (IVC) tumor thrombus occurs in a minority of cases but carries important prognostic implications, underscoring the need for timely and multidisciplinary management. Despite significant perioperative risks and surgical complexity, radical nephrectomy with IVC thrombectomy remains the standard treatment. A structured, multidisciplinary preoperative approach has been reported to improve outcomes. This study aims to evaluate the multidisciplinary team (MDT) surgical approach in the management of RCC with IVC thrombus and its impact on clinical outcomes.

Methods

This retrospective study included all adult patients who underwent radical nephrectomy with IVC thrombectomy in the Uro-Oncology Unit between January 2012 and February 2024. All cases were managed by the surgical MDT following a comprehensive preoperative review. Assessed outcomes included 30-day mortality, operative time, blood loss, perioperative complications, and oncological outcomes.

Results

Fourteen patients (mean age: 52.1 ± 9.8 years; 92.86% male) were included. Most had a Mayo level II thrombus. The 30-day mortality rate was 0%, and Clavien-Dindo class III-IV complications occurred in two patients. The mean operative time was 360 ± 121.2 minutes, and the median intraoperative blood loss was 3000 mL. No significant differences were observed in operative time (p = 0.31) or blood loss (p = 0.55) between lower and higher thrombus levels. The median hospital and ICU stays were 12 and six days, respectively. Over a median follow-up of 19.5 months, the overall survival (OS) rate was 78.57%, and cancer-specific survival (CSS) was 92.86%. Although OS was lower in patients with metastasis, the difference was not statistically significant. Eight patients developed new metastases during follow-up.

Conclusion

The surgical MDT approach in the management of RCC with IVC tumor thrombus was associated with improved perioperative outcomes. These findings highlight the potential benefits of MDT involvement in managing this complex condition and support further investigation in larger, comparative studies.

## Introduction

Renal cell carcinoma (RCC) accounts for approximately 3% of all cancers [1] and is particularly notable for its association with tumor thrombus formation, characterized by tumor extension into blood vessels [2]. Approximately 4-10% of RCC cases present with tumor thrombus extending into the renal vein or inferior vena cava (IVC) at diagnosis [3]. The presence of IVC tumor thrombus is a well-established poor prognostic factor. Untreated cases have a median life expectancy of five months and a one-year disease-specific survival rate of 29% [4].

Radical nephrectomy combined with IVC thrombectomy remains the primary treatment for RCC with IVC thrombus. Surgical resection improves one-year survival rates to 90% in patients without metastases and 60% in those with metastatic disease. Despite these benefits, the procedure carries substantial perioperative morbidity and mortality. Data from the International Renal Cell Carcinoma-Venous Thrombus Consortium (IRCC-VTC) report a 30-day mortality rate of 1.8% [5,6], while other studies report rates ranging from 5% to 10% [7,8]. The level of tumor thrombus is also a predictor of perioperative complications, particularly those classified as high-grade [5,6].

Performing radical nephrectomy with IVC thrombectomy often necessitates vascular clamping. In more advanced cases, extended hepatic mobilization, the Pringle maneuver, and clamping of the hepatoduodenal ligament may be required [9,10]. Mayo level IV thrombi demand even more complex procedures involving cardiopulmonary bypass and possible reconstruction of the right atrium. The surgical complexity is further amplified by the involvement of multiple organ systems, often beyond the urologist's standard scope of practice. Procedures such as high IVC control and hepatic mobilization require advanced surgical techniques and collaboration across specialties.

A surgical multidisciplinary team (MDT) approach is essential for the effective planning and execution of complex cases, such as RCC with IVC tumor thrombus. This coordinated strategy aims to reduce perioperative complications and enhance oncological outcomes [9]. However, detailed descriptions of MDT structures and their impact on outcomes remain limited in the literature.

This study presents our surgical MDT approach to managing RCC with IVC thrombus through radical nephrectomy and thrombectomy, and it highlights the impact of this strategy on perioperative complications, morbidity, and oncological outcomes.

## Materials and methods

Study design and setting

This study is a retrospective observational study conducted at Hamad Medical Corporation (HMC), the main tertiary referral and academic medical center in Qatar. It aims to evaluate the clinical, surgical, and oncological outcomes of patients with RCC and tumor thrombus involving the IVC.

Eligible participants were adult patients (aged ≥18 years) with confirmed RCC and IVC tumor thrombus, which was classified according to the Mayo classification [11], ranging from Mayo level I to IV. All patients underwent radical nephrectomy with thrombectomy by surgical MDT between January 1, 2012, and February 1, 2024. Patients were excluded if they had benign renal masses, lacked IVC involvement, or had incomplete medical or surgical records.

Data were extracted through a structured review of the electronic medical record system. The dataset included demographic information (age, sex), tumor characteristics (side, size, thrombus level), imaging findings, surgical details (operative time, estimated blood loss, use of cardiopulmonary bypass, vascular reconstruction), postoperative complications, and perioperative outcomes (ICU stay, 30-day morbidity and mortality).

Oncological outcomes were also assessed, including tumor histopathology (type, grade), lymph node involvement, presence of metastases, cancer-specific survival (CSS), and overall survival (OS).

Ethical considerations

The study protocol was approved by the Institutional Review Board (IRB) of HMC (approval no. MRC-01-24-369). A waiver of informed consent was granted due to the retrospective nature of the study. All research activities were conducted in accordance with the Declaration of Helsinki, Good Clinical Practice (GCP), and the regulatory requirements of the Ministry of Public Health (MOPH) in Qatar.

MDT approach

All cases were initially discussed in a dedicated oncology MDT meeting, which included senior consultants in uro-oncology, medical oncology, and radiology. For patients selected for surgery, a specialized surgical MDT, comprising uro-oncology and vascular surgery consultants, reviewed each case to determine the surgical strategy. When necessary, additional input was obtained from thoracic or hepatobiliary surgeons.

Subsequently, the case was jointly reviewed with the anesthesia team to finalize the operative plan, including patient positioning, incision type, anticipated blood loss, transfusion needs, potential use of cardiopulmonary bypass, and ICU admission for postoperative monitoring.

Surgical procedure

All procedures were performed under general anesthesia with endotracheal intubation. A midline laparotomy was performed to access the abdominal cavity.

Right-Sided Tumors

The Cattell-Braasch maneuver was employed to medially mobilize the right colon and duodenum, providing exposure to the retroperitoneal structures, including the right renal vein, aorta, and the IVC from the iliac bifurcation to the hepatic veins.

Left-Sided Tumors

The Mattox maneuver was used to mobilize the left colon, spleen, and pancreas to expose the aorta and left renal hilum. Kocherization of the duodenum and mobilization of the hepatic flexure were also performed to allow proximal IVC control near the liver.

Control of the IVC was achieved using vessel loops placed both inferior to the renal veins and proximally at the level of the liver. Both renal veins were isolated and looped. The renal artery was dissected and ligated or clamped early to reduce intraoperative bleeding.

An en bloc resection of the kidney, renal vein, and tumor thrombus was performed. Reconstruction of the IVC was achieved either by primary closure or, when required, using a polytetrafluoroethylene (ePTFE) graft.

Patients with a Mayo level IV thrombus extending into the right atrium underwent radical right nephrectomy with resection of the atrial wall and venous tumor thrombus. Cardiopulmonary bypass and partial atrial wall reconstruction using a bovine pericardial patch were performed.

Postoperative care

All patients were routinely admitted to the ICU postoperatively for close monitoring, hemodynamic stabilization, and early detection of complications.

Statistical analysis

Continuous variables with a normal distribution were presented as mean ± standard deviation (SD), while non-normally distributed variables were reported as median and range. Normality was assessed using the Shapiro-Wilk test. Kaplan-Meier analysis was used to estimate OS and CSS, and survival differences between groups were compared using the log-rank test. A p-value < 0.05 was considered statistically significant.

## Results

Baseline demographic and clinical characteristics

The study included a total of 14 patients who underwent radical nephrectomy with IVC thrombectomy for RCC with varying levels of IVC tumor thrombus. All procedures were performed by a surgical MDT. Demographic and clinical characteristics are summarized in Table 1. The mean age was 52.1 ± 9.8 years, with most patients being male and presenting with right-sided tumors. At diagnosis, 14.29% had distant metastases, whereas 28.57% had lymph node involvement. Detailed individual patient characteristics, surgical parameters, and outcomes are provided in the supplementary table (Table 4).

**Table 1:** Patient demographics at diagnosis.

Characteristics	Results
Mean age (years), mean ± SD	52.1 ± 9.8
Sex, n (%)	
Male	13 (92.86%)
Female	1 (7.14%)
Laterality, n (%)	
Right	8 (57.14%)
Left	6 (42.86%)
Metastasis at diagnosis, n (%)	
Yes	2 (14.29%)
No	14 (85.71%)
Lymph node involvement at diagnosis, n (%)	
Yes	4 (28.57%)
No	10 (71.43%)

Tumor characteristics

The mean radiological and pathological tumor sizes were 10.47 ± 2.92 cm and 10.64 ± 2.76 cm, respectively. The majority of patients (64.29%) had level II tumor thrombus based on the Mayo classification. Clear cell RCC was the most common histological subtype (78.57%), and most tumors were graded as ISUP (International Society of Urological Pathology) grade III or IV. Table 2 outlines detailed tumor characteristics, including thrombus level, histological subtype, and grade.

**Table 2:** Tumor characteristics. * Tumor thrombus level based on Mayo classification. RCC: renal cell carcinoma; ISUP: International Society of Urological Pathology

Characteristics	Results
Radiological tumor size (cm), mean ± SD	10.47 ± 2.92
Pathological tumor size (cm), mean ± SD	10.64 ± 2.76
Tumor thrombus level, n (%) *	
Level I	2 (14.29%)
Level II	9 (64.29%)
Level III	2 (14.29%)
Level IV	1 (7.14%)
Histopathological type, n (%)	
Clear cell RCC	11 (78.57%)
Chromophobe RCC	1 (7.14%)
Papillary RCC	1 (7.14%)
Unclassified RCC	1 (7.14%)
Histopathological ISUP grade, n (%)	
Grade II	1 (7.14%)
Grade III	7 (50.00%)
Grade IV	6 (42.86%)

Surgical and perioperative outcomes

Three patients (21.43%) underwent preoperative renal artery embolization: one for active bleeding and two for bleeding risk mitigation. All surgeries were performed by a multidisciplinary team involving urologists and vascular surgeons. The hepatobiliary team assisted in two cases requiring extended liver mobilization.

Primary IVC reconstruction was carried out in all cases except one, which required ePTFE graft placement. One patient with a right-sided RCC and a Mayo level IV IVC thrombus extending into the right atrium underwent radical nephrectomy, thrombectomy, and partial atrial wall resection with repair using a bovine pericardial patch under cardiopulmonary bypass.

The mean operative time for the entire cohort was 360 ± 121.2 minutes. When comparing lower-level IVC thrombus (Mayo levels I and II) to higher-level thrombus (Mayo levels III and IV), the mean operative times were 377.7 ± 118.7 minutes and 295 ± 130 minutes, respectively. An independent samples t-test showed no statistically significant difference in operative time between the two groups (p = 0.31). The mean difference was 82.73 minutes (95% CI: −88.59 to 254.04).

The median intraoperative blood loss for the entire cohort was 3000 mL. When analyzed by tumor thrombus level, there was no significant difference in blood loss between patients with lower-level IVC thrombus (Mayo levels I and II) and those with higher-level thrombus (Mayo levels III and IV), with median values of 3600 mL and 3000 mL, respectively. This difference was not statistically significant (p = 0.55).

All patients were routinely admitted to the ICU postoperatively for close monitoring and observation as part of the planned perioperative protocol.

Two patients experienced Clavien-Dindo class III-IV complications: one patient developed gastrointestinal bleeding, while the other required a second-look laparotomy for postoperative hemorrhage and subsequently developed ischemic hepatitis and deep vein thrombosis. No 30-day mortality was observed. Table 3 summarizes the surgical and perioperative details.

**Table 3:** Surgical and perioperative outcomes.

Characteristics	Results
Preoperative embolization, n (%)	
Yes	3 (21.43%)
No	11 (78.57%)
Operative time (min), mean ± SD	360 ± 121.2
Intraoperative blood loss (mL), median, range	3000 (500–12000)
ICU stay (days), median, range	6 (1–26)
Hospital stay (days), median, range	12 (3–32)
30-day mortality, n (%)	0 (0%)
Postoperative complication, (Clavien-Dindo class), n (%)	
Class 0–II	1
Class III–IV	2

Survival outcomes

**Figure 1:**
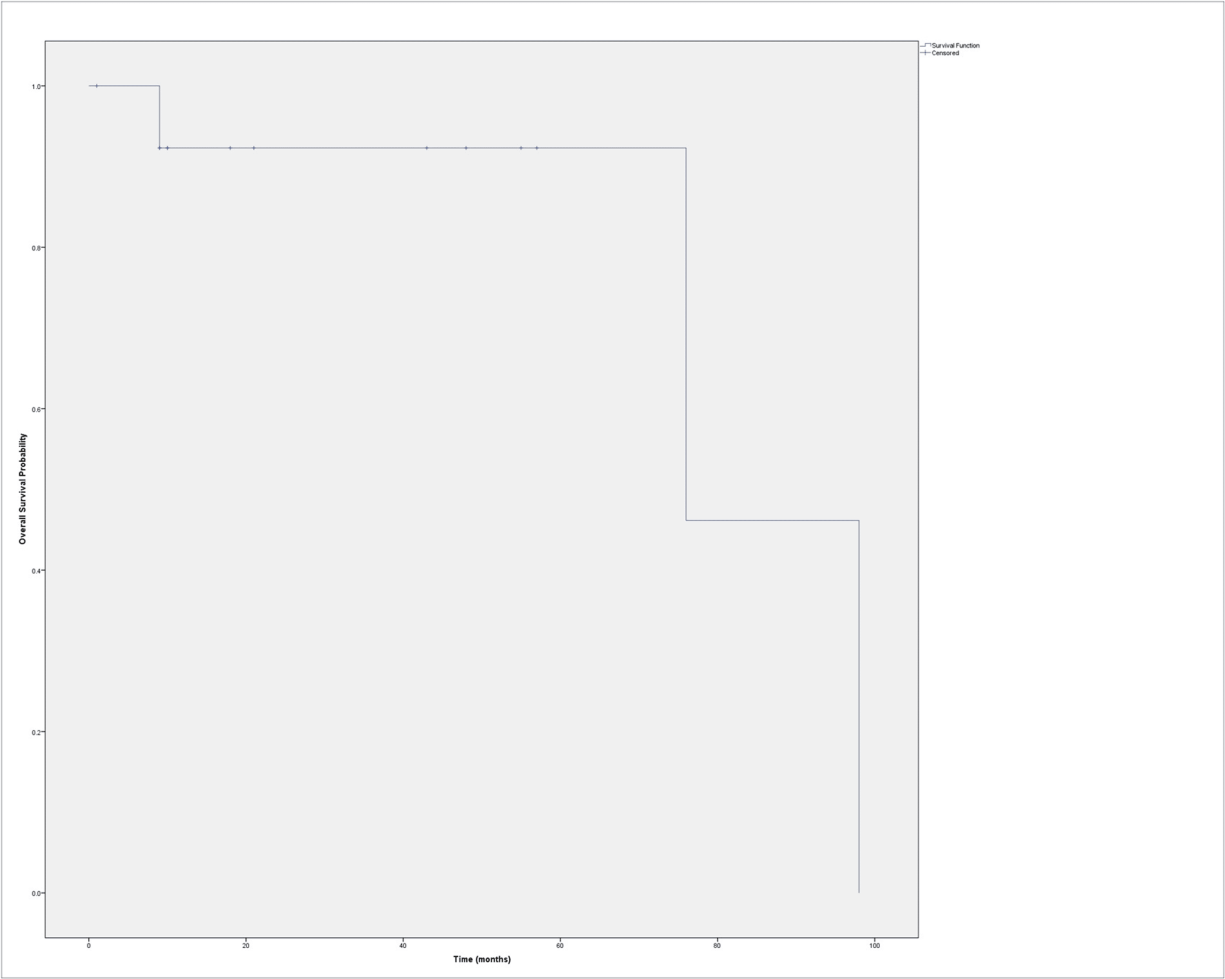
Kaplan–Meier curve showing overall survival (in months) following radical nephrectomy and IVC thrombectomy in the entire patient cohort. Censored cases are indicated by plus signs (+). IVC: inferior vena cava

At a median follow-up of 19.5 months (range: 1-98), the OS rate was 78.57%, while the CSS rate was 92.86%. Figure 1 illustrates the Kaplan-Meier curve for OS. Among the three deaths recorded, two were due to non-cancer causes (respiratory failure and aortic dissection), and one was attributed to cancer progression in a patient who presented with metastasis at the time of surgery.

**Figure 2:**
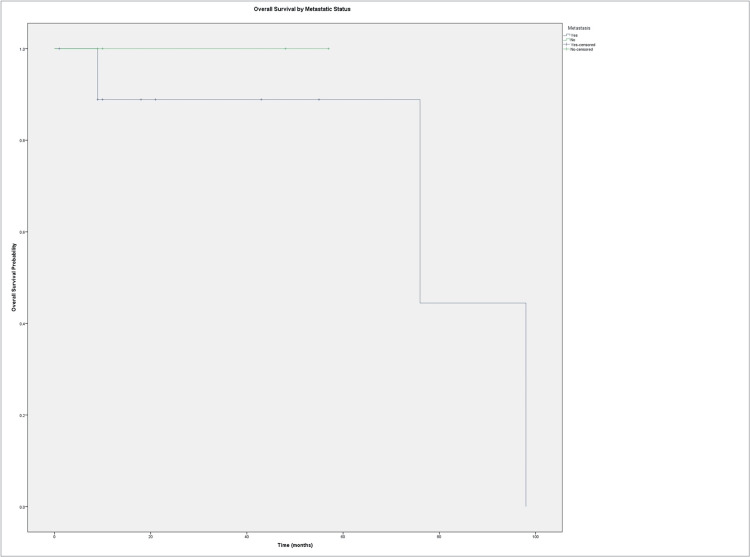
Kaplan–Meier curve of overall survival stratified by metastatic status.

Kaplan-Meier analysis indicated that patients with metastasis had lower OS compared to patients without metastasis, although this difference was not statistically significant (log-rank p = 0.505) (Figure 2).

Metastasis and recurrence

**Figure 3:**
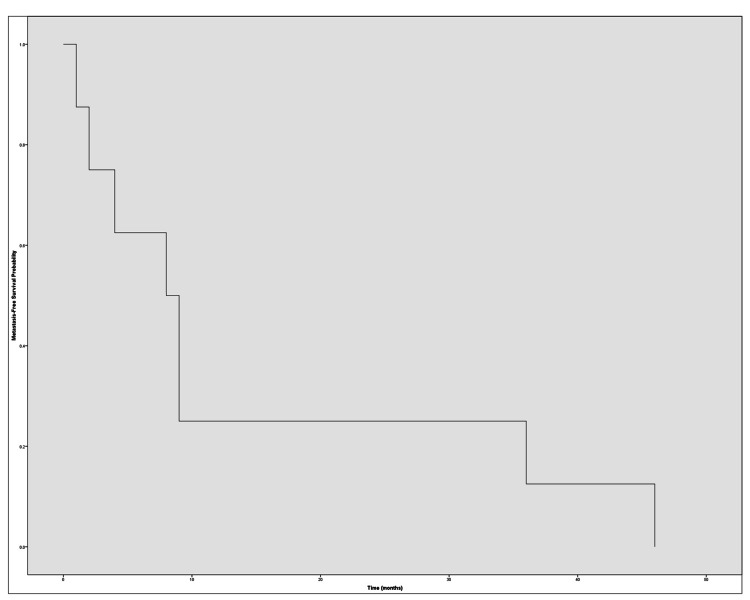
Kaplan–Meier curve illustrating metastasis-free survival over time (in months), excluding the two patients who presented with metastases at diagnosis.

Two patients presented with metastatic disease at the time of diagnosis. During follow-up, eight additional patients developed distant metastases, with a median time to metastasis of 8.5 months (range: 1-46 months). The most frequent metastatic sites were the spine (n=5), lungs (n=4), and liver (n=2). Additionally, two patients experienced local recurrence in the surgical bed, both in conjunction with distant metastases. A total of eight patients (57.14%) received adjuvant systemic therapy, including tyrosine kinase inhibitors, immune checkpoint inhibitors, or combination regimens. Metastasis-free survival was assessed using Kaplan-Meier analysis, excluding the two patients with metastasis at presentation (Figure 3).

## Discussion

In this study, we report the outcomes of surgical MDT management for cases of RCC with IVC thrombus. Neoplastic extension into the renal vein or IVC occurs in approximately 4% to 10% of cases, while cardiac chamber involvement is observed in around 1% [3,12]. Surgical excision remains the gold standard, as chemotherapy and radiotherapy have shown limited efficacy [12]. Immunotherapy, used in neoadjuvant or adjuvant settings, may reduce perioperative morbidity and improve recurrence-free and progression-free survival [12-15]; however, it has not replaced the need for surgery.

The MDT approach has been associated with favorable outcomes in localized, locally advanced, and metastatic RCC [9,16,17]. It also facilitates timely, patient-centered decision-making and comprehensive care planning [18]. Radical nephrectomy with IVC thrombus removal is a technically demanding procedure, often requiring gastrointestinal and liver mobilization, control of hepatic blood flow, and, in cases of intracardiac extension, cardiopulmonary bypass. These complexities contribute to a perioperative morbidity rate of up to 70% and mortality rates ranging from 3% to 16% [10-12,19]. Given that these surgeries often exceed the capacity of a single specialty, the MDT surgical approach is indispensable [9].

In our cohort, the surgical MDT strategy showed promising outcomes. There was no 30-day mortality, even in high-complexity cases, while the complication rate remained low. The median hospital stay was six days, significantly shorter than the 10-20 days reported in other series [9,20,21]. Two critical surgical planning challenges further underscore the importance of this model. In one patient, a retro-aortic left renal vein draining into the IVC at the level of the left iliac vein was missed during preoperative planning, leading to difficult intraoperative bleeding control and increased blood loss. In another case, the thrombus level was underestimated, necessitating an intraoperative revision of the surgical plan. These incidents highlight the importance of detailed preoperative imaging, particularly with radiologist input, to anticipate anatomical variations and enhance surgical preparedness.

When comparing intraoperative parameters, the mean operative time in our cohort was 360 minutes. This duration was shorter than in some open surgery series [9], comparable to others [22], and slightly longer than in robotic or laparoscopic cohorts, possibly due to the fact that those studies included only lower-level (I-II) thrombi [23,24].

Regarding oncological outcomes, the recurrence rate was relatively high. Excluding the two patients with metastases at diagnosis, eight patients developed new metastases during follow-up. Nonetheless, the OS rate was 78.57%, and CSS was 92.86% at a median follow-up of 19.5 months. These results are consistent with the natural history of locally advanced RCC and emphasize the prognostic importance of complete, aggressive surgical resection [4,5,9-12].

This study has several limitations. It is retrospective in design and based on a relatively small cohort, which limits both the statistical power and the generalizability of the findings. Additionally, as with many retrospective analyses, the study is constrained by the availability and completeness of recorded data, which may limit the depth of certain outcome analyses. However, the low case volume also reflects the rarity of RCC with IVC tumor thrombus, particularly in the context of increased detection of early-stage RCC through widespread imaging. Despite these constraints, the study offers valuable insights into the potential benefits of an MDT approach in managing complex RCC cases, particularly with respect to perioperative safety and oncological outcomes.

The surgical management of RCC with IVC tumor thrombus is inherently complex and associated with significant perioperative risk. In this single-arm study, the implementation of a surgical MDT approach was associated with improved operative efficiency, lower perioperative morbidity and mortality, and shorter ICU and hospital stays. These findings highlight the potential role of MDT strategies in enhancing patient outcomes and suggest that such collaborative approaches should be further explored in future prospective studies.

## Conclusions

This study demonstrates that a surgical MDT approach to RCC with IVC tumor thrombus is associated with improved perioperative outcomes. We recommend adopting routine MDT involvement in the surgical management of these cases to enhance perioperative planning, reduce complications, and improve overall care. While further research is needed to confirm these findings, MDT involvement appears to offer a promising strategy for optimizing outcomes in these challenging cases.

## Appendices

**Table 4:** Summary of individual patient characteristics and surgical outcomes for RCC with IVC tumor thrombus managed by the surgical MDT. RCC: renal cell carcinoma; IVC: inferior vena cava; MDT: multidisciplinary team; TNM: tumor, node, metastasis; CPB: cardiopulmonary bypass; ICU: intensive care unit; mL: milliliters

Case number	Age (years)	Sex	Laterality	TNM stage	Thrombus level	Surgical teams involved	CPB used	Operation time (minutes)	Blood loss (mL)	Post-operation ICU stay (days)	Post-operation hospital stay (days)	Adjuvant immunotherapy	Follow-up duration (months)	Status at last follow-up
1	67	Female	Right	pT4 N0 M0	4	Urology, vascular, cardiac	Yes	420	5500	2	5	Sunitinib	98	Death
2	49	Male	left	pT3 N1 M0	2	Urology, vascular, hepatobiliary	No	590	6000	3	14	None	10	Alive
3	45	Male	Right	pT3b N1 M1	2	Urology, vascular	No	230	1200	1	4	None	9	Death
4	48	Male	Right	pT3 N1 M0	2	Urology, vascular	No	325	8000	2	4	None	1	Alive
5	57	Male	Right	pT3b N0 M0	2	Urology, vascular	No	220	1000	2	4	Sunitinib	57	Alive
6	62	Male	Right	pT3b N0 M0	3	Urology, vascular, Hepatobiliary	No	305	3000	2	3	None	55	Alive
7	48	Male	Right	pT3b N0 M0	3	Urology, vascular	No	160	500	1	4	Sunitinib	76	Death
8	30	Male	Left	PT3b N1 M0	2	Urology, vascular	No	480	12000	7	12	Sunitinib & Pembrolizumab	21	Alive
9	54	Male	Left	pT3b N0 M0	1	Urology, vascular	No	365	1000	2	8	Axitinib & Pembrolizumab	48	Alive
10	52	Male	Left	pT3b N0 M0	2	Urology, vascular	No	450	5000	5	27	Pembrolizumab	43	Alive
11	66	Male	Left	pT3b N0 M0	2	Urology, vascular	No	300	1000	3	7	Pembrolizumab	9	Alive
12	57	Male	Right	pT3b N0 M0	2	Urology, vascular	No	510	6000	26	32	Pembrolizumab	18	Alive
13	42	Male	Left	pT3b N0 M0	1	Urology, vascular	No	390	1000	4	7	Sunitinib	10	Alive
14	52	Male	Right	pT3b N0 M0	2	Urology, vascular	No	295	3600	6	12	None	9	Alive
